# Supplementary material for: Robustness and parameter geography in post-translational modification systems
Source: PLoS Comput Biol. 2020 May 4;16(5):e1007573. doi: 10.1371/journal.pcbi.1007573 (PMC7224580; doi:10.1371/journal.pcbi.1007573)
Supplement: S1 Appendix — This document provides a comprehensive description of our implementations of the methods described in the paper and guidelines for navigating the supplemental code and datasets. Supplemental figures: (A) Workflow for computing and parsing solutions with Paramotopy. Supplemental tables: (A) Seeds used to initialise the MATLAB pseudo-random number generator for sampling; (B) Seeds used to initialise the MATLAB pseudo-random number generator for VEGAS sampling; (C) Details of the refined connectivity graphs. (PDF) [file pcbi.1007573.s001.pdf]

S1 Appendix: Supplemental Methods

Kee-Myoung Nam[1], Benjamin M. Gyori[2], Silviana V. Amethyst[3], Daniel J. Bates[4], and Jeremy Gunawardena[1]

[1]Department of Systems Biology, Harvard Medical School, Boston, MA, USA
[2]Laboratory of Systems Pharmacology, Harvard Medical School, Boston, MA, USA
[3]Department of Mathematics, University of Wisconsin–Eau Claire, Eau Claire, WI, USA
[4]Department of Mathematics, United States Naval Academy, Annapolis, MD, USA

Abstract

This note provides supporting information for “Robustness and parameter geography in post-translational modification systems.” It should be read in conjunction with the paper, which provides further details.

Contents

A remark on notation. . . . 3

1 Dataset availability **3**

2 Computing solutions with Paramotopy **3**

Running Paramotopy. . . . 4

Resolving path failures. . . . 6

Generating the ILR samples. . . . 6

Naming the input files. . . . 7

Parsing the Paramotopy output. . . . 7

Recomputing solutions with more stringent Bertini settings. . . . 8

Gathering solutions across re-runs. . . . 10

Classifying solutions. . . . 11

3 Certifying solutions with alphaCertified **12**

The general method. . . . 12

Uncertifiable solutions. . . . 15

4 Generating VEGAS samples **17**

5 Building and refining the connectivity graph **20**

Building the initial connectivity graphs. . . . 20

Refining the connectivity graphs. . . . 22

6 Locating blinking points within the connectivity graph **24**

7 Computing the K-fold visibility ratio **25**

8 Plotting the figures in the main text **26**

A remark on notation. We label files and directories in the supplemental datasets with blue monospaced font (e.g., `sigma1_N1_log_part1_posreal_run0`), and scripts in **S1 Code** in green monospaced font (e.g., `run_default_sharpen.sh`). We use indices in square brackets to denote a family of paths varying with respect to the index (e.g., `sample_N1_[J]_log`, with `J` running over $1,\ldots,40$, refers to the family of paths `sample_N1_1_log`, `sample_N1_2_log`, and so on).

1 Dataset availability

All data generated throughout our analysis are available on Mendeley Data, under the DOIs provided in **S1 File**. These data files have been organized into 251 datasets, which can be grouped into six categories:

- **Input files (Dataset 1)**: All Paramotopy input files used in our analysis (§2).
- **Sample files (Datasets 2A, 2B, ...)**: All Paramotopy sample files used in our analysis, including those for the Paramotopy re-runs (§2).
- **Configurations files (Dataset 3)**: All configurations files used to specify Bertini settings for Paramotopy re-runs (§2).
- **Solutions files (Datasets 4A.1, 4A.2, ...)**: All posreal counts files, statistics files, final posreal counts files, proper solutions files, and bistable points files generated from raw Paramotopy output (§2).
- **Certification files (Datasets 5A.1, 5A.2, ...)**: All composite polynomials files, composite solutions files, and composite summary files generated through certifying the solutions obtained with Paramotopy with alphaCertified, along with the raw alphaCertified output for the uncertifiable solutions (§3).
- **Connectivity files (Dataset 6)**: All MAT files specifying the initial and refined connectivity graphs of the bistable region for each value of σ (§5).

Details on each of these datasets are provided in the corresponding section of this document.

2 Computing solutions with Paramotopy

Here, we provide an overview of the workflow we used to compute solutions to Eq. 10 with Bertini (version 1.5) and Paramotopy (version 1.0.3.3) [1, 2],[1] along with guidelines for navi-

[1]The vast majority of the computations were performed with Bertini version 1.5 and Paramotopy version 1.0.3.3 on the Orchestra cluster, which was retired on March 31, 2018; a small number of Paramotopy re-runs were performed with Bertini version 1.5.1 and Paramotopy version 1.0.3.5 on the O2 cluster. Neither Bertini nor Paramotopy exhibited any significant differences in behavior or output between the two versions. The summary metadata file (see §2, *Running Paramotopy*), `metadata.tsv` (**S1 Dataset**), indicates the versions of Bertini and Paramotopy used for each Paramotopy run and re-run in our analysis.

gating the supplemental code and datasets. All of the following computations were performed on the Harvard Medical School Orchestra and O2 research computing clusters (cf. footnote 1; see also the Materials and Methods). Sample generation and downstream data processing were performed with Python (version 3.6.0) and MATLAB (version R2016b), as described below. A visual summary of this workflow is given in Fig. A.

Running Paramotopy. To run Paramotopy to solve a polynomial system on a set of parameter points, two text files are necessary:

- A *sample file*, which enumerates the parameter points at which the polynomial system is to be solved; and
- An *input file*, which specifies the polynomial system, the path to the sample file, and the values of any additional constants in the system (in our case, the conserved quantities σ, λ, and ζ).

Each sample file follows a format in which the first line specifies the number of parameter points in the sample, and each subsequent line specifies a parameter point, as follows:[2]

```
250000
9.33800023 0 4.951382248 0 0.8191515255 0 0.226647373 0 0.1044532314 0 1.538546311 0 1.516123949 0
↪  5.805465284 0
1.758719585 0 4.47224637 0 2.240754659 0 2.361857076 0 0.3468770687 0 2.190015678 0 1.899227327 0
↪  1.374745685 0
1.131055897 0 5.999210239 0 0.2055436739 0 0.123058971 0 3.999828164 0 0.5425741084 0 0.4357257068 0
↪  0.1814724675 0
1.224630225 0 0.7358534335 0 0.53225864 0 6.698002977 0 4.970483648 0 1.842852163 0 0.2026463679 0
↪  1.364747879 0
1.149915127 0 7.907821956 0 0.8921887943 0 0.4532788927 0 3.338804068 0 0.1665797119 0 1.815942776 0
↪  1.110281152 0
...
```

Each parameter point is specified with complex-valued coordinates, i.e., the first value refers to the real part of the first parameter, the second value to the imaginary part of the first parameter, the third value to the real part of the second parameter, and so on.

Each input file assumes the following format:[3]

```
2 1 8 3
zeta*u*v + alpha*v^2 + beta*zeta^2*u^2 + (alpha*epsilon0 - alpha*sigma*epsilon0 - alpha +
↪  phi1*zeta)*u*v^2 + (epsilon1*zeta - sigma*epsilon1*zeta - zeta + beta*phi2*zeta^2)*u^2*v +
↪  (beta*epsilon2*zeta^2 - beta*sigma*epsilon2*zeta^2 - beta*zeta^2)*u^3 + alpha*phi0*v^3 -
↪  (alpha*epsilon0 + phi1*zeta)*u^2*v^2 - (epsilon1*zeta + beta*phi2*zeta^2)*u^3*v -
↪  beta*epsilon2*zeta^2*u^4 - alpha*phi0*u*v^3
zeta*u*v + alpha*v^2 + beta*zeta^2*u^2 + (alpha*phi0 - alpha*lambda*phi0 - alpha)*v^3 + (phi1*zeta -
↪  lambda*phi1*zeta - zeta + alpha*epsilon0)*u*v^2 + (beta*phi2*zeta^2 - beta*lambda*zeta^2*phi2 -
↪  beta*zeta^2 + epsilon1*zeta)*u^2*v + beta*epsilon2*zeta^2*u^3 - (alpha*epsilon0 +
↪  phi1*zeta)*u*v^3 - (epsilon1*zeta + beta*phi2*zeta^2)*u^2*v^2 - beta*epsilon2*zeta^2*u^3*v -
↪  alpha*phi0*v^4
u, v
```

[2]See **Dataset 2A**, sample_N1_1_log.

[3]See **Dataset 1**, sigma1_N1_log_part1.in.

```
constant sigma,lambda,zeta;
sigma = 1;
lambda = 1;
zeta = 1;
1
/n/groups/gunawardena/chris_nam/parameter_geography/samples/sample_N1_1_log
alpha 0.1 0 10 0 3
beta 0.1 0 10 0 3
epsilon0 0.1 0 10 0 3
epsilon1 0.1 0 10 0 3
epsilon2 0.1 0 10 0 3
phi0 0.1 0 10 0 3
phi1 0.1 0 10 0 3
phi2 0.1 0 10 0 3
```

The first eight lines in the above example specify the polynomial system (lines 2 and 3; cf. Eq. 10) and the variables (line 4), as well as the identities and values of the constants (lines 5 to 8). Line 9 indicates the use of an externally defined set of parameter points, and line 10 specifies the path to the sample file. The remaining lines specify an ordering for the parameters, which determines how the values in the sample file are interpreted as they are read. (The numbers following each parameter, which determine a mesh of parameter points to be used in the absence of a sample file, are not significant in the context of our analysis.)

Now, running Paramotopy on these files amounts to simply calling (in the same directory as the input file), e.g.,

```
$ paramotopy input.in
```

where `input.in` is the name of the input file; then entering a series of numeric commands via the interactive Paramotopy interface to perform the computations. Details on this interface are given in the Paramotopy manual [3]. As our analysis required efficiently solving Eq. 10 at many parameter points and values of σ, we implemented a shell script, **`run_default_sharpen.sh`**, that automates these commands. In particular, this script directs Paramotopy to sharpen each solution to 25 digits with post-endgame iterations of Newton's method (`SharpenOnly`: 1 and `SharpenDigits`: 25).

The output from Paramotopy is written to a directory with the prefix `bfiles_` in the same directory as the input file. For instance, the Paramotopy output for an input file with the name `input.in` would be written to the directory `bfiles_input.in/`. Within this directory are subdirectories for each Paramotopy "run" on the given input file, named `run0/`, `run1/`, and so on, which contains output from distinct calls to Paramotopy on the given input file, possibly with edits to the input file (e.g., to point to a different sample file), different Bertini settings, different choice of Step 1 point, and other choices of the user's discretion. The raw output directories generated for every Paramotopy run we performed in our analysis, including the re-runs described below (Materials and Methods; see also §2, *Recomputing solutions with more stringent Bertini settings*), are available upon request to the authors.

The Bertini settings used for each run are determined by an XML file,[4] which are stored in an

[4]This XML file contains not only the Bertini settings with which Paramotopy performs the run, but also other metadata essential to running Paramotopy, such as the paths to the Bertini and Paramotopy executables, the number of cores with which to perform Step 2, and so on.

external directory named `.paramotopy/`, located in the user's home directory by default. Upon running Paramotopy on an input file, e.g., `input.in`, for the first time, Paramotopy copies a pre-existing *default settings file*, `.paramotopy/defaultprefs.xml`, to a new settings file unique to the input file, `.paramotopy/input.inprefs.xml`. Subsequently, each Paramotopy run on `input.in` parses the settings in the latter settings file, and further copies this file to the corresponding run directory, as `prefs.xml`. Changes to the Bertini settings, via the Paramotopy interface, are reflected by changes to both copies of the XML file.

The default settings file used for our analysis, which contain the first-pass setting values given in Table 3, is included in **S1 Dataset** as **`defaultprefs.xml`**.[5] Furthermore, the Bertini settings used across all Paramotopy runs in our analysis are tabulated in a tab-delimited file, **`metadata.tsv`** (**S1 Dataset**), which was generated by parsing each `prefs.xml` file within each run directory, via the script **`write_metadata.py`**.

Resolving path failures. As described in the Materials and Methods, Paramotopy automatically collects all path failures—instances of premature termination in path tracking—that arise during Step 2, which can then be "resolved" with further iterations of parameter homotopy continuation. The script **`run_default_sharpen.sh`** performs one iteration of path failure resolution, with the first-pass Bertini settings given in Table 3. However, one iteration is sometimes insufficient to resolve all path failures. In these cases, we resolved all remaining path failures by manually performing additional iterations of path failure resolution, via the Paramotopy interface, while changing the Bertini settings as necessary. All instances of such manual intervention are enumerated in the summary metadata file, **`metadata.tsv`** (**S1 Dataset**), with each iteration of path failure resolution labeled with a separate set of Bertini settings.

Generating the ILR samples. We implemented a MATLAB script, **`getLogSample.m`**, that generates an independent logarithmic random (ILR) sample of 10^7 points in $\mathcal{H} = [0.1, 10]^8$, and writes these points to 40 separate Paramotopy sample files, each containing 2.5×10^5 points. These sample files are available in **Dataset 2A** as **`sample_N1_[J]_log`**, where **`J`** runs over $1, \ldots, 40$.

We implemented a second MATLAB script, **`getLogExternalSample.m`**, that generates four ILR samples of 10^6 points, one in each of the boxes, $\mathcal{H}_p = [10^{-p}, 10^p]^8$, for $p = 2, 3, 4, 5$. Each of these samples were then written to four Paramotopy sample files, each containing 2.5×10^5 points. These sample files are available in **Dataset 2A** as **`sample_N[P]_[J]_log`**, where **`P`** refers to the exponent, p, and **`J`** runs over 1, 2, 3, 4.

Finally, we also implemented a Python script, **`collapse_sample.py`**, that takes as input a sample file with parameter points in $\mathbb{C}^8$, and generates a new sample file with the fifth and sixth coordinates excised. This script was used to generate, for 32 of the 56 sample files described above (**Dataset 2A**, **`sample_N[P]_[J]_log`**, with **`J`** $= 1, \ldots, 16$ for **`P`** $= 1$ and **`J`** $= 1, \ldots, 4$ for **`P`** > 1), a corresponding sample file (**Dataset 2A**, **`sample_N[P]_[J]_log_6D`**) with parameter points in $\mathcal{H}_p^* = [10^{-p}, 10^p]^6$, for $p = 1, \ldots, 5$. These six-dimensional sample files are available in **Dataset 2A**.

[5] This file does not contain the values given in Table 3 for `SharpenDigits` (25) and `SharpenOnly` (1); these values were incorporated via the script, **`run_default_sharpen.sh`**, as mentioned above.

The real part of each coordinate of each sampled parameter point was specified to 10 significant figures.[6] The seeds used to initialize the MATLAB pseudo-random number generator are given in Table A.[7]

Naming the input files. Each first-pass Paramotopy input file was named according to the format `sigma[S]_[SUFFIX].in`, in which `S` refers to the value of σ indicated in the input file (lines 6 and 7), and `SUFFIX` contains some description for the associated sample file(s). For instance, the input file **`sigma1_N2_log.in`** (**Dataset 1**) was used to solve Eq. 10 at each parameter point in the ILR sample of 10^6 points in $\mathcal{H}_2$, with $\sigma = 1.0$; the computation was performed over four runs, one for each of the sample files, **`sample_N2_[J]_log`** with **`J`** $= 1, 2, 3, 4$ (**Dataset 2A**). Input files pointing to six-dimensional sample files were labeled with the modifier `michaelis-menten` (e.g., **Dataset 1**, **`sigma1_michaelis-menten_N2_log.in`**), while all input files pointing to VEGAS sample files (see below) were labeled with the modifier `vegas` (e.g., **Dataset 1**, **`sigma1_vegas_N1_log_part1.in`**). A list of all input file–sample file correspondences in our analysis is given in the summary metadata file, **`metadata.tsv`** (**S1 Dataset**), and the first-pass input files used in our analysis are available in **Dataset 1**.

As described in the Materials and Methods, we re-ran Paramotopy on any parameter points for which a first-pass Paramotopy run failed to yield the expected number of "proper" solutions (meaning nonzero, finite, and non-singular solutions; see the Materials and Methods). Given a completed first-pass Paramotopy run, `bfiles_sigma[S]_[SUFFIX].in/run[R]/`, the associated *re-run input files* were named `sigma[S]_[SUFFIX]_retest[T]_run[R].in`, where `T` $= 0, 1, 2, \ldots$ is the re-run iteration. A list of all re-run input files used in our analysis, with their corresponding *re-run sample files* (see below), is provided in the summary metadata file, **`metadata.tsv`** (**S1 Dataset**), and the re-run input files used in our analysis are also available in **Dataset 1**.

Parsing the Paramotopy output. The output for each Paramotopy run contains three text files, in particular:

- `nonsingular_solutions0`, which lists the non-singular solutions for each parameter point;
- `singular_solutions0`, which lists the singular solutions for each parameter point; and
- `failed_paths0`, which lists any unresolved path failures for each parameter point.

We implemented a Python script, **`gather_paramotopy_data.py`**, that parses the first two files and counts the number of small, ambiguous, infinite, insufficiently precise, nonzero real, nonzero non-real, and positive real non-singular solutions per parameter point, according to the classification in Eq. 31, as well as the number of singular solutions per parameter point. (The script

[6] Any coordinate specified with fewer than 10 significant figures possesses implicit trailing zeros.

[7] One should edit **`getLogSample.m`** and **`getLogExternalSample.m`** with the appropriate seeds to replicate the samples.

accepts user-specified values for the numerical thresholds T_{zmin}, T_{zmax}, T_∞, and T_d.) From this, the script generates four output files:

- A text file in which each line contains a parameter point, followed by the corresponding number of positive real solutions (*posreal counts file*);
- A new sample file containing all parameter points with at least one small, ambiguous, infinite, singular, or insufficiently precise solution (*re-run sample file*);
- A text file containing summary statistics over the sample, along with a logarithmic histogram of solution values (*statistics file*); and
- A PDF file with a visualization of the histogram (*histogram file*).

To streamline the generation of these output files in the context of our analysis, we implemented an accompanying shell script, **`gather_paramotopy_data.sh`**, which determines the paths to which the above output files are written for a given Paramotopy run. Specifically, given a completed first-pass Paramotopy run, `bfiles_sigma[S]_[SUFFIX].in/run[R]/`, this script uses the following names for the four output files:

- The posreal counts file: `sigma[S]_[SUFFIX]_posreal_run[R]`;
- The re-run sample file: `sample_retest_sigma[S]_[SUFFIX]_0_run[R]`;
- The statistics file: `sigma[S]_[SUFFIX]_stats_run[R]`; and
- The histogram file: `sigma[S]_[SUFFIX]_stats_run[R].pdf`.

In addition, **`gather_paramotopy_data.sh`** also parses the `failed_paths0` file for any unresolved path failures, the presence of which can confound the classification of solutions;[8] and automatically generates a new Paramotopy input file (*re-run input file*) that points to the re-run sample file, named `sigma[S]_[SUFFIX]_retest0_run[R].in`.

The re-run input files and re-run sample files generated throughout our analysis are available in **Datasets 1** and **2B**, respectively. The posreal counts files and statistics files generated throughout our analysis are available within a collection of 48 datasets, as indicated in **S1 File**. Specifically, **Dataset 4**$x.y$ contains either the posreal counts files ($y = 1$) or statistics files ($y = 3$) for a particular value of σ indicated by the letter x, from $x = \mathbf{A}$ ($\sigma = 1.0$), $x = \mathbf{B}$ ($\sigma = 1.0078125$) and so on, to $x = \mathbf{X}$ ($\sigma = 5000$). See **S1 File** for details.

Recomputing solutions with more stringent Bertini settings. As mentioned above, we re-ran Paramotopy on any parameter points for which a first-pass Paramotopy run did not yield the expected number of proper solutions. Every first-pass Paramotopy run yielded at least one such parameter point, and so was followed by at least one re-run. Each iteration of this procedure involved two steps:

[8] If any unresolved path failures exist, then the script merely prompts the user to resolve them; we resolved all such path failures manually, via the Paramotopy interface, as described above (see §2, *Resolving path failures*).

1. Run Paramotopy on the re-run input file, pointing to the corresponding re-run sample file, with new Bertini settings; and

2. Parse the output with `gather_paramotopy_data.py` and `gather_paramotopy_data.sh`.

To facilitate the first step, we implemented a shell script, `run_retest_config.sh`, that runs Paramotopy on the new input file in a manner similar to `run_default_sharpen.sh`. The main difference between the two scripts is that `run_retest_config.sh` parses a second text file, a *configurations file*, which enumerates the Step 2 Bertini settings to be employed in the re-run. An example configurations file reads as follows:[9]

```
CONDNUMTHRESHOLD        1e+17
FINALTOL        1e-11
MAXNUMBERSTEPS        20000
SHARPENDIGITS        30
SHARPENONLY        1
TRACKTOLBEFOREEG        1e-08
TRACKTOLDURINGEG        1e-09
```

As mentioned above (see §2, *Running Paramotopy*), a complete list of the re-runs we performed in our analysis and the Bertini settings used for each re-run—not only in Step 2, but also in Step 1 and each iteration of path failure resolution—is given in the summary metadata file, `metadata.tsv` (**S1 Dataset**). In addition, **Dataset 3** contains all configurations files used in our analysis. To summarize:

- For all re-runs on points sampled from $\mathcal{H}_1 = \mathcal{H}$, $\mathcal{H}_2$, $\mathcal{H}_3$, or $\mathcal{H}_4$ (ILR or VEGAS), we specified a set of Bertini settings for each value of the exponent, $p = 1, 2, 3, 4$, and retest iteration, $t = 0, 1, 2, \ldots$, in configurations files named `config_retest[T]_N[P]_log.txt`, with `T` $= t$ and `P` $= p$.

- For all re-runs on points sampled from $\mathcal{H}_1^* = \mathcal{H}^*$, $\mathcal{H}_2^*$, or $\mathcal{H}_3^*$ (ILR or VEGAS), we specified a set of Bertini settings for each value of the exponent, $p = 1, 2, 3$, and retest iteration, $t = 0, 1, 2, \ldots$, in configurations files named

  `config_retest[T]_michaelis-menten_N[P]_log.txt`

  with `T` $= t$ and `P` $= p$.

- For re-runs on points (ILR-)sampled from $\mathcal{H}_5$, $\mathcal{H}_4^*$, or $\mathcal{H}_5^*$, we followed the same strategy for many earlier re-run iterations ($t \leq 6$ for $\mathcal{H}_5$, $t \leq 8$ for $\mathcal{H}_4^*$, and $t \leq 10$ for $\mathcal{H}_5^*$), specifying the Bertini settings in configurations files named

  `config_retest[T]_N5_log.txt`
  `config_retest[T]_michaelis-menten_N4_log.txt`
  `config_retest[T]_michaelis-menten_N5_log.txt`

[9] See **Dataset 3**, `config_retest0_N1_log.txt`.

with T $= t$. For many of the later re-run iterations ($t \geq 6$ for $\mathcal{H}_5$, $t \geq 5$ for $\mathcal{H}_4^*$, and $t \geq 4$ for $\mathcal{H}_5^*$), we alternatively specified the Bertini settings in configurations files unique to the re-run iteration itself, named

config_retest[T]_sigma[S]_N5_log_run[R].txt
config_retest[T]_sigma[S]_michaelis-menten_N4_log_run[R].txt
config_retest[T]_sigma[S]_michaelis-menten_N5_log_run[R].txt

for appropriate values of S, T, and R. (Any re-run for which a configurations file of the second type was not specified was performed with one of the first type.)

The output of these re-runs was then processed in the same way as the first-pass runs, using gather_paramotopy_data.py and gather_paramotopy_data.sh. Given a completed re-run under bfiles_sigma[S]_[SUFFIX]_retest[T]_run[R].in/run0/, we used the following naming scheme for the output files generated by gather_paramotopy_data.sh:

- The posreal counts file: sigma[S]_[SUFFIX]_retest[T]_posreal_run[R];
- The (new) re-run sample file: sample_retest_sigma[S]_[SUFFIX]_[T+1]_run[R];
- The statistics file: sigma[S]_[SUFFIX]_retest[T]_stats_run[R];
- The histogram file: sigma[S]_[SUFFIX]_retest[T]_stats_run[R].pdf; and
- The (new) re-run input file: sigma[S]_[SUFFIX]_retest[T+1]_run[R].in.

As with the output files described in the previous section, these files are also available in **Datasets 1**, **2B**, and **4x.y**, for $x = \mathbf{A}, \ldots, \mathbf{X}$ and $y = 1, 3$. See **S1 File** for details.

Gathering solutions across re-runs. Once a sequence of successive re-runs results in an empty re-run sample file—at which point a complete set of proper solutions has been identified for every parameter point in a given sample file—the Paramotopy output across all of these re-runs must be parsed to obtain a definitive set of proper solutions for each parameter point. To this end, we implemented a Python script, gather_all_solutions.py, which runs through the re-runs in reverse order, parses each nonsingular_solutions0 file, and writes the solution sets for each parameter point in the original (first-pass) sample file to a text file (*proper solutions file*) with the same format as a nonsingular_solutions0 file. Each proper solutions file can then be parsed in much the same way as any nonsingular_solutions0 file, using gather_paramotopy_data.py, to generate the corresponding *final posreal counts file*. We parsed these final posreal counts files to finally determine a definitive subset of bistable points in each sample file, from which we proceeded to estimate bistable volumes and perform the downstream analyses delineated in the following sections.

Given a first-pass Paramotopy run, bfiles_sigma[S]_[SUFFIX].in/run[R]/, for which all required re-runs were completed, the corresponding proper solutions file is available in one of 118 datasets, as indicated in **S1 File**, at the path sigma[S]_[SUFFIX]_solutions_run[R].

Each of these datasets, **Dataset 4x.y**, was named to correspond to a single value of `S` (x = **A**,...,**X**) and one or more values of `SUFFIX` ($y = 4, 5, \ldots$). For example, **Dataset 4A.4** contains all proper solutions files generated from the Paramotopy runs,

```
bfiles_sigma1_N1_log_part1.in/run[R]/
bfiles_sigma1_N1_log_part2.in/run[R]/
bfiles_sigma1_N1_log_part3.in/run[R]/
bfiles_sigma1_N1_log_part4.in/run[R]/
bfiles_sigma1_N1_log_part5.in/run[R]/
```

The final posreal counts file generated by parsing `sigma[S]_[SUFFIX]_solutions_run[R]` in **Dataset 4x.y** is available in **Dataset 4x.2**, at the path `sigma[S]_[SUFFIX]_posreal_run[R]`. See **S1 File** for details.

Classifying solutions. As mentioned above and in the Materials and Methods, we used the classification in Eq. 31 to classify the non-singular solutions obtained with Paramotopy as small, ambiguous, infinite, insufficiently precise, nonzero real, or nonzero non-real. For every first-pass Paramotopy run, we performed this classification with $T_{\text{zmax}} = 10^{-10}$, $T_{\text{zmin}} = 10^{-25}$, $T_{\infty} = 10^8$, and $T_d = 20$ (Materials and Methods). The following considerations dictated our choices for these thresholds for the re-runs:

- We set $T_{\text{zmax}} = 10^{-10}$ for all re-runs except those for which manual inspection of the `nonsingular_solutions0` file revealed highly sharpened solutions with real or imaginary parts with absolute value less than 10^{-10}, in which case T_{zmax} was decreased appropriately.

- Similarly, we set $T_{\infty} = 10^8$ for all re-runs except those for which either manual inspection of the `nonsingular_solutions0` file revealed highly sharpened solutions with real or imaginary parts with absolute value exceeding 10^8, or a large number of such solutions were found with `gather_paramotopy_data.py`. (The latter was often the case with re-runs involving parameter points sampled from $\mathcal{H}_5$, $\mathcal{H}_3^*$, $\mathcal{H}_4^*$, or $\mathcal{H}_5^*$.)

- We set $T_{\text{zmin}} = 10^{-25}$ for virtually all re-runs, with the only exception afforded to late re-runs ($t \geq 9$) involving parameter points sampled from $\mathcal{H}_5^*$. The re-runs involving these points were particularly computationally expensive, with each re-run requiring several days to complete on as many as 20 cores, despite only running on thousands of points. In view of these difficulties, we set $T_{\text{zmin}} = T_{\text{zmax}}$ for re-runs with $t \geq 9$ involving these points, with T_{zmax} set as described above.

- In contrast to T_{zmax}, T_{zmin}, and T_{∞}, we incrementally increased T_d for each successive re-run, so as to reflect increases in the Bertini setting, `SharpenDigits`. We adhered to the general rule of setting $T_d = \texttt{SharpenDigits} - 5$, except for re-runs with $\texttt{SharpenDigits} > 65$, in which case T_d was set to 55 (for re-runs involving points sampled from $\mathcal{H}_5^*$) or 60 (for re-runs involving points sampled from $\mathcal{H}_4^*$).

A complete list of the threshold values used for each Paramotopy run and re-run can be found under the directory `thresholds/` in **S1 Dataset**.

3 Certifying solutions with alphaCertified

As described in the Materials and Methods, the software package alphaCertified [4] can be used to verify, or *certify*, that each numerical solution reported by Paramotopy is indeed an approximation of a true (non-singular) solution to Eq. 10. Here, we describe our usage of alphaCertified to certify randomly selected subsets of the solutions we obtained with Paramotopy.

The general method. Briefly, alphaCertified requires three text files as input:

- A *polynomials file*, which specifies the polynomial system of interest with *rational* coefficients;
- A *solutions file*, which enumerates the non-singular solutions to be certified; and
- A *configurations file*, which enumerates values for the alphaCertified settings.

In contrast with Paramotopy, alphaCertified does not allow the use of free parameters in specifying the polynomial system. Moreover, the coefficients in the polynomials file must be specified rationally (i.e., as fractions of integers) [5]. Therefore, for each parameter point and corresponding solution set, a polynomials file and solutions file must be separately generated and passed into alphaCertified.

As with Bertini, the details of the computations underlying the certification procedure can be modulated by introducing user-specified values for the alphaCertified settings, via the configurations file [5]. Of particular importance among the alphaCertified settings are `ALGORITHM`, which specifies the choice of certification algorithm to be run; and `PRECISION`, which specifies the minimum floating-point precision with which the certification is performed.

While most non-singular solutions reported by Bertini or Paramotopy should be certifiable as they are, some reported solutions may require some sharpening, with additional iterations of Newton's method, prior to certification. alphaCertified facilitates this sharpening with the `NEWTONONLY` setting, which, if set to 1, forces alphaCertified to sharpen the reported solutions in lieu of certifying them; the sharpened solutions are then written to a second solutions file, which can be passed into alphaCertified as input for a second certification attempt. As long as the initial set of reported solutions are reasonably accurate, this procedure can be repeated as many times as necessary to certify every solution in the set.

To streamline the *en masse* certification of solution sets obtained with Paramotopy, we implemented a Python script, `certify_paramotopy_output.py`, that applies this iterative certify-and-sharpen procedure to each solution set in a given proper solutions file (see §2, *Gathering solutions across re-runs*). For each solution set, this script generates the requisite alphaCertified input files; runs alphaCertified up to a maximum number of iterations to certify each solution in the set; and writes relevant portions of the alphaCertified output to an in-memory stream before deleting the output files. Upon running through every solution set, the script then dumps the contents of the stream to three composite output files—a *polynomials file*, a *solutions file*, and a *summary file*—that provide a comprehensive overview of the certification results. The

user can also direct the script to randomly choose a subcollection of the solution sets and certify the solutions in only those chosen.

For each proper solutions file generated through the procedure described in §2, we used `certify_paramotopy_output.py` to randomly choose a 5% subcollection of solution sets in the file, without replacement, and certify each solution in each chosen solution set. Up to five iterations of certification and four iterations of sharpening were performed per solution set, and the first certification attempt was performed with a minimum floating-point precision of 128 bits (`PRECISION`: 128). Whenever a certification attempt yielded an uncertified solution, every solution in the set was sharpened with two iterations of Newton's method (`NEWTONONLY`: 1 and `NUMITERATIONS`: 2), and the minimum floating-point precision (`PRECISION`) to be used by alphaCertified was quadrupled[10] for the next certification attempt.

The composite output files thus generated are organized among 39 datasets, as indicated in **S1 File**. Specifically, the composite output files generated by certifying solutions obtained at $\sigma = 1.0$ chosen from the proper solutions file, `sigma1_[SUFFIX]_solutions_run[R]`, are available at the paths

```
sigma1_[SUFFIX]_polynomials_run[R]
sigma1_[SUFFIX]_solutions_run[R]
sigma1_[SUFFIX]_summary_run[R]
```

in **Dataset 5A.**y, where $y = 1, \ldots, 16$ corresponds to a different set of values for `SUFFIX`. For example, **Dataset 5A.1** contains all composite output files generated from certifying the solutions chosen from the proper solutions files (**Dataset 4A.4**)

```
sigma1_N1_log_part1_solutions_run[R]
sigma1_N1_log_part2_solutions_run[R]
sigma1_N1_log_part3_solutions_run[R]
sigma1_N1_log_part4_solutions_run[R]
sigma1_N1_log_part5_solutions_run[R]
```

Similarly, the composite output files generated by certifying the solutions obtained at $\sigma > 1.0$ in the proper solutions file, `sigma[S]_[SUFFIX]_solutions_run[R]`, for `S` $= \sigma$, are available at the paths

```
sigma[S]_[SUFFIX]_polynomials_run[R]
sigma[S]_[SUFFIX]_solutions_run[R]
sigma[S]_[SUFFIX]_summary_run[R]
```

in **Datasets 5B** through **5X**, where the letter corresponds to a different value of `S`. See **S1 File** for details.

The most informative of the three composite output files is the summary file, which assumes the following format:[11]

[10] This choice reflects the quadratic convergence of Newton's method near non-singular solutions [1, 4].
[11] See **Dataset 5A.1**, `sigma1_N1_log_part1_summary_run0`.

```
SEED        5466950

## CERT3
PRECISION        128
RANDOMSEED        1527404473
NCERTIFIED        7
NDISTINCT        7

-2.7807466222427254321763569113100000000006e-1 -1.5777218104420236108234571305700000000004e-30
-6.1103589541428358982068074119399999999999e-1 -3.1554436208840472216469142611299999999996e-30
1
3.757792005201672e-28
5.273881619722980e-30
7.125286982454219e1

5.3365317836835818083179315416799999999986e-1 -3.1554436208840472216469142611299999999996e-30
7.4237522089761618802123488531300000000014e-1 -6.3108872417680944432938285222599999999992e-30
1
2.147061136950063e-28
8.934547116032614e-30
2.403100133746304e1

2.6733556585702930804874823925200000000002e-1 0
-4.3434335710478942087345155750000000000002e-1 -3.1554436208840472216469142611299999999996e-30
1
1.074267566861614e-27
3.887088867849576e-30
2.763681519470696e2

1.1766306874952466117797646341200000000000e-1 -6.7626206778356529060707186714899999999998e-1
6.9638034361694429249107965565800000000006e-1 5.0182793693556765896928721129300000000004e-1
1
1.194358351639590e-27
1.250375390627320e-29
9.551998228630952e1

5.7205496096042706533399519027600000000003e-1 -7.4409422469138958335308557469399999999999e-1
-3.9840296874840208628957566390200000000006e-1 3.7311380895520275450470277943400000000005e-1
1
4.966778998599759e-27
1.016514700836792e-29
4.886086737861364e2

1.1766306874952466117797646340900000000000e-1 6.7626206778356529060707186713599999999997e-1
6.9638034361694429249107965567100000000007e-1 -5.0182793693556765896928721129300000000004e-1
1
9.560334891608861e-28
1.000872766386505e-29
9.551998228630952e1

5.7205496096042706533399519028900000000004e-1 7.4409422469138958335308557471899999999998e-1
-3.9840296874840208628957566391400000000005e-1 -3.7311380895520275450470277944700000000006e-1
1
1.206479843166171e-26
2.469214952361338e-29
4.886086737861364e2


## CERT12
...
```

Running through each section in this excerpt:

1. The first line contains the seed used to initialize the Python pseudo-random number generator, which was used to randomly choose the 12500 solution sets to be certified in each proper solutions file. This line also heads the other two composite output files.

2. The certification results for each solution set is headed by a line starting with ##; the indicated number is the index of the solution set in the given proper solutions file.

3. The next two lines indicate the minimal floating-point precision used by alphaCertified and the seed used to initialize the alphaCertified pseudo-random number generator.

4. The next two lines indicate the number of certified solutions in the set, and the number of *distinct* certified solutions in the set.

5. In each of the following blocks of six lines, the first two lines provide a single solution in the set;[12] the third line indicates whether the solution was certified; and the last three lines provide the three α-theoretic constants that alphaCertified computed to perform the certification [4].

Parsing these summary files with another script, `count_certified_solutions.py`, we found that every solution was successfully certified in all but a tiny minority of the chosen solution sets (Materials and Methods; see also §3, *Uncertifiable solutions*). In particular, this procedure successfully certified every solution in every chosen solution set that corresponded to a parameter point sampled from the full eight-dimensional parameter space (i.e., from $\mathcal{H}_p$, for $p = 1, \dots, 5$) or from the six-dimensional boxes $\mathcal{H}_1^*$, $\mathcal{H}_2^*$, $\mathcal{H}_3^*$, and $\mathcal{H}_4^*$.

Uncertifiable solutions. As mentioned above and in the Materials and Methods, a tiny minority of the chosen solution sets, all corresponding to parameter points sampled from $\mathcal{H}_5^*$, exhibited at least one uncertifiable solution. Of the 1.05×10^6 solution sets chosen from the 84 proper solutions files generated from Paramotopy runs on ILR-sampled points from $\mathcal{H}_5^*$, 106 (~0.01%) solution sets exhibited at least one uncertifiable solution. 99 out of these 106 solution sets exhibited exactly one uncertified solution. As such, we believe these uncertifiable solutions exert, in all likelihood, a negligible effect on the accuracy of the bistable volume estimates given in Fig. 9.

We have included the raw alphaCertified input and output files for each of these 106 solution sets in **Dataset 5Z**. These files are organized within directories named

`sigma[S]_michaelis-menten_N5_log_uncertified_run[R]/`

which contain, along with alphaCertified configurations files for each iteration of certification and sharpening, a polynomials file, `poly[J]`, and a solutions file, `sols[J]`, for each solution set

[12]Note that each solution in this set was reported with fewer than $\lfloor 128(\log 2) \rfloor = 38$ digits in the proper solutions file (**Dataset 4A.4**, `sigma1_N1_log_part1_solutions_run0`), and so each solution is here padded with additional trailing digits.

(with index J) with an uncertifiable solution. The corresponding alphaCertified output is stored within the subdirectory

sigma[S]_michaelis-menten_N5_log_uncertified_run[R]/cert[J]/

which is organized into further subdirectories, containing alphaCertified output for each iteration of certification (`iter0/`, `iter1/`, etc.) and sharpening (`refine0/`, `refine1/`, etc.). Details on these output files are given in the alphaCertified manual [5].

Manual inspection of this output suggests that the uncertifiable solutions are in fact close to singular. This is illustrated by the following uncertifiable solution at $\sigma = 500$ for solution set 114206, obtained at the parameter point,

$$(\alpha = 1.324820725 \times 10^{-5},\ \beta = 542.3801621,\ \varepsilon_0 = 1.379194063 \times 10^{-5},$$
$$\varepsilon_1 = 2.573853535 \times 10^{-5},\ \varphi_1 = 8.255586243 \times 10^{-5},\ \varphi_2 = 10792.23839)\,,$$

in the proper solutions file (**Dataset 4U.5**)

sigma500_michaelis-menten_N5_log_solutions_run0

for which the other four solutions were successfully certified:[13]

```
...
-------------------------
Point 3
-2.9915859670531327869171640615971612419 40e-1 -1.5324404074654801962743184768012438467 50e-2
4.665616213314700125588759966206408371477e10 -8.318475801841183664369618835997359446115e7
Approx solution: No
...
-------------------------
Point 3
-2.546292388103373089717332570633095289[...]e-1 7.743457243220775040799787097425987410[...]e-2
4.347759679373646112105612239451018861[...]e10 -1.849816782711876699596826454445276665[...]e9
Approx solution: No
...
-------------------------
Point 3
-3.450511500779821510179509470309470386[...]e-1 -8.072789218643111756213171795973223087[...]e-2
4.720348881178225317220316946309835906[...]e10 -1.266175347928749014037521022624021896[...]e9
Approx solution: No
...
-------------------------
Point 3
-2.539456820672542690747017879314331862[...]e-1 -7.724107252686608220802841205767736411[...]e-2
4.240795346194117759142437023680425001[...]e10 -4.042541342176611526017400770396955808[...]e9
Approx solution: No
...
-------------------------
Point 3
```

[13] Each block (delimited by ellipses) is an excerpt from the alphaCertified output file (**Dataset 5Z**)

sigma500_michaelis-menten_N5_log_uncertified_run0/cert114206/iter[K]/summary

with **K** running over $0, \ldots, 4$. The bracketed ellipses in each solution coordinate denote additional digits that were truncated for clarity.

```
-1.043237231174485682560888106094532999[...]e-2 -9.609310054975773349573136953632851506[...]e-2
2.755303217148915895425684175344250323[...]e10 -2.720684249387080880977728373637908438[...]e10
Approx solution: No
...
```

Note that neither the real nor imaginary parts of either solution coordinate exhibits any kind of convergence over four iterations of sharpening. This is in stark contrast to the following solution at $\sigma = 500$ for the same parameter point, which required one additional iteration of sharpening to be certified (cf. footnote 13):

```
...
------------------------
Point 1
2.711697293037518652559727467647141533246e-3 -4.786511379749346170411739081581957650622e-3
6.423470449717805036265457071732250297725e6 1.146777955413306243144732602889882180284e7
Approx solution: No
...
------------------------
Point 1
2.711697293037518652559727467647141533261[...]e-3 -4.786511379749346170411739081581957650645[...]e-3
6.423470449717805036265457071732250297704[...]e6 1.146777955413306243144732602889882180285[...]e7
Approx solution: Yes
...
------------------------
Point 1
2.711697293037518652559727467647141533261[...]e-3 -4.786511379749346170411739081581957650645[...]e-3
6.423470449717805036265457071732250297704[...]e6 1.146777955413306243144732602889882180285[...]e7
Approx solution: Yes
...
------------------------
Point 1
2.711697293037518652559727467647141533261[...]e-3 -4.786511379749346170411739081581957650645[...]e-3
6.423470449717805036265457071732250297704[...]e6 1.146777955413306243144732602889882180285[...]e7
Approx solution: Yes
...
------------------------
Point 1
2.711697293037518652559727467647141533261[...]e-3 -4.786511379749346170411739081581957650645[...]e-3
6.423470449717805036265457071732250297704[...]e6 1.146777955413306243144732602889882180285[...]e7
Approx solution: Yes
...
```

This suggests that each of these uncertifiable solutions corresponds to a path-tracking endpoint with an ill-conditioned Jacobian (albeit not so ill-conditioned for it to be deemed singular) which exhibited the correct endgame convergence behavior as required by Bertini, and yet does not approximate a true non-singular solution. One way to recover the "missing" solutions may be to simply re-run Paramotopy with yet more stringent Bertini settings, including a smaller value for `FinalTol`.

4 Generating VEGAS samples

Here, we describe our implementation of VEGAS importance sampling. The core of the algorithm described in the Materials and Methods was implemented as a MATLAB function,

getSamplesVegas(), as given in the file getSamplesVegas.m. In short, getSamplesVegas() performs a single iteration of Step 2a in the algorithm: Given an existing set of bistable points, $\widehat{\mathcal{M}}_\sigma^{(t)}$, and eight partitions of the interval $[0.1, 10]$, $\{I_i^{(j)} : i = 1, \ldots, M; j = 1, \ldots, 8\}$, the function updates the bin lengths in each partition according to the bin frequencies, and returns a new sample, $\mathcal{S}^{(t+1)}$, of parameter points (Materials and Methods).

To initialize the VEGAS sampling procedure for each value of σ, we implemented a MATLAB script, getFirstLogVegasSample.m, which performs Step 1 of the algorithm—initializing the partitions, $\{I_i^{(j)}\}$, so that each bin has the same length in logarithmic coordinates—and calls getSamplesVegas() on these bins and the subset of parameter points ILR-sampled from $\mathcal{H}$ that exhibit bistability at the given value of σ. Each such set of bistable points was read from a concatenation of the posreal counts files generated from all Paramotopy runs on these ILR-sampled points at the given value of σ (*aggregate posreal counts file*), which is available at the path[14] (**Dataset 4*x*.2**, for $x =$ **J**, …, **U**)

sigma[S]_N1_log_posreal_total

Essential metadata concerning the sampling procedure—the sample size per iteration, N; the iteration number, $t = 0$; the smoothing factor, K; and the updated bins, $\{I_i^{(j)}\}$—were stored in a MAT file for future access. We then performed Step 2b of the algorithm by following the workflow in Fig. A, obtaining a new set of bistable points, $\mathcal{M}_\sigma \cap \mathcal{S}^{(1)}$. We then augmented the previous bistable set with these points, obtaining the set $\widehat{\mathcal{M}}_\sigma^{(1)}$ and completing one iteration of the algorithm.

To repeat Step 2, we implemented a second MATLAB script, getNthLogVegasSample.m, that is essentially identical to getFirstLogVegasSample.m except that it reads the bistable points obtained from the previous iteration (i.e., $\widehat{\mathcal{M}}_\sigma^{(1)}$), as well as the information in the aforementioned MAT file, and redirects these data into getSamplesVegas(). In this way, we were able to obtain a successively nested sequence of bistable sets, $\widehat{\mathcal{M}}_\sigma^{(2)}, \ldots, \widehat{\mathcal{M}}_\sigma^{(T)}$, up to some maximum number of iterations, T.

For each value of σ in Eq. 14, we performed the above procedure for $T = 6$ iterations with $M = 50$ bins, generating $N = 10^6$ points per iteration with a smoothing factor of $K = 1000$. Each VEGAS sample of 10^6 points were written to four sample files, each containing 2.5×10^5 points. These files can be found at the paths

sample_vegas_N1_log_sigma[S]_[T]_[P]

[14]More specifically, for each value of σ, we concatenated all the posreal counts files named (**Dataset 4*x*.2**, $x =$ **J**, …, **U**)

sigma[S]_N1_log_part[P]_posreal_run[R]

with S $= \sigma$, over all admissible values of P and R. (For instance, for $\sigma = 2.5$, $x =$ **J**, P runs over $1, \ldots, 6$, and R over $0, \ldots, 3$; for $\sigma = 10$, $x =$ **O**, P runs over $1, \ldots, 4$, and R over $0, \ldots, 3$. See the summary metadata file, metadata.tsv (**S1 Dataset**), for a complete list of these runs, and see **S1 File** for details on where these files are available.) The resulting aggregate posreal counts files are located at the paths (**Dataset 4*x*.2**)

sigma[S]_N1_log_posreal_total

See **S1 File** for details.

in **Datasets 2C** through **2N**, for `T` $= t-1 = 0,\dots,5$ and `P` $= 1,\dots,4$. See **S1 File** for details.

To perform Step 2b for each value of σ and t, we followed the workflow in Fig. A with four Paramotopy input files, one for each of the four sample files. The input files were named `sigma[S]_vegas_N1_log_part[P].in` (**Dataset 1**), with `S` $= \sigma$ and `P` $= 1,\dots,4$; the Paramotopy output for different iterations of the sampling, $t = 1,\dots,6$, were organized into different Paramotopy runs, `run0/` through `run5/`. Upon completing each run and its associated re-runs, we compiled a definitive set of proper solutions for each parameter point in the proper solutions files (**Dataset 4**x**.4**, for $x =$ **J**,…,**U**; see §2, *Gathering solutions across re-runs* and **S1 File** for details)

`sigma[S]_vegas_N1_log_part[P]_solutions_run[T]`

for the appropriate values of `S`, `P`, and `T`. Each of these proper solutions files was then parsed with `gather_paramotopy_data.py` to generate the final posreal counts file (**Dataset 4**x**.2**, for $x =$ **J**,…,**U**)

`sigma[S]_vegas_N1_log_part[P]_posreal_run[T]`

These final posreal counts files were then concatenated (cf. footnote 14), with `S` $= \sigma$ and `T` $= t-1$ fixed and with `P` varying over $1,\dots,4$, to generate an aggregate posreal counts file enumerating all parameter points sampled from the t-th iteration of the VEGAS sampling procedure (**Dataset 4**x**.2**, for $x =$ **J**,…,**U**):

`sigma[S]_vegas_N1_log_all_posreal_run[T]`

(The bistable subset in each of these aggregate posreal counts files is $\mathcal{M}_\sigma \cap \mathcal{S}^{(t)}$.) The aggregate posreal counts files with `T` $= 0,\dots,t-1$ were then parsed by `getNthLogVegasSample.m`, to generate the next sample, $\mathcal{S}^{(t+1)}$.

We note that we also performed Paramotopy runs for $\sigma = 1.0$ on each of the VEGAS samples described above (Results). More specifically, for each of the aforementioned sample files,

`sample_vegas_N1_log_sigma[S]_[T]_[P]`

we followed the workflow in Fig. A with input file `sigma1_threshold_vegas[S]_part[P].in` (**Dataset 1**), with each iteration of the sampling (`T` $= 0,\dots,5$) corresponding to a different Paramotopy run, `run0/` through `run5/`. Upon completing each run and its associated re-runs, we compiled a definitive set of proper solutions for each parameter point in the proper solutions files (**Dataset 4A.**y, for $y = 16,\dots,27$; see §2, *Gathering solutions across re-runs* and **S1 File** for details)

`sigma1_threshold_vegas[S]_part[P]_solutions_run[T]`

for the appropriate values of `S`, `P`, and `T`; from each of these proper solutions files, we generated the final posreal counts file (**Dataset 4A.2**)

`sigma1_threshold_vegas[S]_part[P]_posreal_run[T]`

with which we found that no points within any of these VEGAS samples exhibit bistability at $\sigma = 1.0$ (Results).

As described in the Results, we generated one additional VEGAS sample ($T = 1$) of $N = 6 \times 10^6$ parameter points from the total bistable set identified for $\sigma = 2.5$ among the 4×10^6 ILR-sampled points and the 6×10^6 VEGAS-sampled points, from which we attempted to identify further bistable points at $\sigma = 1.0, 1.5, 2.0$. To do this, we implemented another MATLAB script, `getLowSigmaLogVegasSample.m`, that runs identically to `getFirstLogVegasSample.m` except that it parses the aggregate posreal counts file obtained by concatenating the files (**Dataset 4J.2**)

`sigma2.5_vegas_N1_log_all_posreal_run[T]`

for `T` = 1,...,6. We used $M = 50$ sub-intervals and a smoothing factor of $K = 1000$ to generate this sample, which was written to 24 Paramotopy sample files of 2.5×10^5 points each. These files can be found at the paths

`sample_vegas_N1_log_lowsigma_[P]`

in **Dataset 2O**, for `P` = 1,...,24. From this, we followed the workflow in Fig. A with 24 Paramotopy input files for $\sigma = 1.0, 1.5, 2.0$; these files were named `sigma[S]_vegas_N1_log_part[P].in` (**Dataset 1**), with `S` = σ and `P` = 1,...,24. As only one iteration of VEGAS sampling was performed in this instance, a single Paramotopy run was completed with each input file.

5 Building and refining the connectivity graph

Building the initial connectivity graphs. The algorithm for computing a spanning forest of the connectivity graph (Materials and Methods) was implemented as a C++ MEX sub-routine, `connectivity.cpp`, which was then compiled into a MEX file and called within a wrapping MATLAB script (`runLogConnectivity.m`). As described in the Materials and Methods, the algorithm requires a user-defined distance threshold, Δ, which we set to 0.15 throughout our analysis. The bistable sets used to construct the initial connectivity graphs (Table 1) for each value of σ listed in Eq. 15 are enumerated in the *aggregate bistable points files*[15] (**Dataset 4*x*.2**, for x = **H**,...,**U**)

[15]For each value of σ, we first generated the aggregate posreal counts file (**Dataset 4*x*.2**, for x = **H**,...,**U**)

`sigma[S]_vegas_N1_log_posreal_total`

by concatenating the aggregate posreal counts files (**Dataset 4*x*.2**)

`sigma[S]_vegas_N1_log_all_posreal_run[T]`

over `T` = 0,...,5. We then generated a *bistable points file* (**Dataset 4*x*.2**),

`sigma[S]_vegas_N1_log_multistable`

containing only those points indicated as bistable in the new aggregate posreal counts file. This was implemented in a simple Python script, `count_bistable.py`. Similarly, the bistable points file (**Dataset 4*x*.2**)

`sigma[S]_N1_log_multistable`

was generated by parsing the aggregate posreal counts file (**Dataset 4*x*.2**)

sigma[S]_combined_vegas_N1_log_multistable

from which runLogConnectivity.m generated two objects as output: a sparse adjacency matrix enumerating the edges in the spanning forest, and a list of labels that indicate, for each vertex, the connected component containing it. These data were written to MAT files, which are included in **Dataset 6** at the path sigma[S]_data/sigma[S]_log_connect.mat.

Probing this data is then straightforward, as shown below:

```
>> load('sigma10_data/sigma10_log_connect.mat');
>> whos
  Name                   Size                      Bytes  Class     Attributes

  A                3236189x3236189              70425936  double    sparse
  components       3236189x1                    25889512  double
  dx                     1x1                           8  double

>> mode(components)                           % Label for the largest component

ans =

     1

>> max(components)                            % Number of components

ans =

      452663

>> sum(components==1)                         % Number of points in the largest component

ans =

     2659036

>> nonlargest = components(components~=1);   % Discard all points within the largest component
>> mode(nonlargest)                           % Label for the second-largest component

ans =

       30566

>> sum(nonlargest==mode(nonlargest))          % Number of points in the second-largest component

ans =

    33

>>
```

Upon loading the MAT file specifying the initial connectivity graph for $\sigma = 10$, we find that the MAT file contains three objects: the adjacency matrix (A), the list of component labels

sigma[S]_N1_log_posreal_total

These two bistable points files were then concatenated to generate the *aggregate bistable point file* (**Dataset 4***x***.2**)

sigma[S]_combined_vegas_N1_log_multistable

which was finally used to build the initial connectivity graph.

(`components`), and the value of Δ used to construct the graph (`dx`). The graph decomposes into 452663 components, the largest of which has label 1 and contains 2659036 points out of 3236189; among the remaining points, the largest component has label 30566 and contains a mere 33 points. Doing this for every initial connectivity graph, for each of the values of σ listed in Eq. 15, we obtain the figures given in Table 1. (We have implemented a MATLAB script, **`reportConnectivityData.m`**, that reports these figures for any MAT file containing the appropriate data.)

Refining the connectivity graphs. Given a connectivity graph that decomposes into components $C_1,\ldots,C_L$ in descending order of size, we refine the graph through an iterative three-step process (Results): (1) generate a new sample of points that "link" each non-largest component to the largest component via straight lines, (2) determine the bistable subset of this new sample with Bertini and Paramotopy, and (3) compute a new connectivity graph with these new bistable points. We implemented the first step as a MATLAB function, `getLinkSamples()`, as given in the file **`getLinkSamples.m`**. This function uses a MATLAB class [6] that wraps the C++ Approximate Nearest Neighbor (ANN) library [7, 8] to identify, for a randomly selected point $\theta^{(i)}$ in the i-th non-largest component in the connectivity graph, K "approximate nearest neighbors" $v^{(1)},\ldots,v^{(K)}$ in the largest component. These points are approximate nearest neighbors in the sense that, if $\mu^{(j)}$ is the true j-th nearest neighbor to $\theta^{(i)}$ in the largest component, then $v^{(j)}$ is an $(1+\varepsilon)$-approximation of $\mu^{(j)}$ with respect to distance from $\theta^{(i)}$:

$$d(v^{(j)},\theta^{(i)}) \le (1+\varepsilon)\, d(\mu^{(j)},\theta^{(i)}),$$

for some approximation factor $\varepsilon > 0$ that can be made arbitrarily small. For each of these neighbors, the function then identifies $\lfloor d(v^{(j)},\theta^{(i)}) / 0.98\Delta \rfloor$ points along the straight line between $v^{(j)}$ and $\theta^{(i)}$, by jumping in increments of 0.98Δ starting from $\theta^{(i)}$. Doing this for each of the $L-1$ non-largest components, we obtain a "linking sample" of $\sum_{i=2}^{L}\sum_{j=1}^{K} \lfloor d(v^{(j)},\theta^{(i)}) / 0.98\Delta \rfloor$ parameter points, from which we can identify a subset of new bistable points, using the workflow in Fig. A, to refine the connectivity graph. We can then repeat this procedure, until either the graph consists of a single component, or a desired number of iterations has been completed.

We implemented a wrapping script, **`getNewLogLinkSample.m`**, that loads a given MAT file specifying a connectivity graph and runs `getLinkSamples()` to generate a linking sample. We used an approximation factor of $\varepsilon = 0.001$ throughout our analysis (Results), and set K as (Materials and Methods)

$$K \leftarrow \begin{cases} j+1 & \text{if } \#C_2 > 10 \\ 3\left(j+1\right) & \text{if } 3 < \#C_2 \le 10 \\ 10\left(j+1\right) & \text{otherwise.} \end{cases}$$

where $j = 0,1,2,\ldots$ is the iteration number. The resulting linking sample, $\mathcal{S}$, was then written to $\lceil \#\mathcal{S} / \left(2.5\times 10^5\right)\rceil$ Paramotopy sample files, each containing 2.5×10^5 points. These files are included in **Dataset 2P**, as **`sample_log_link_sigma[S]_[J]_[P]`**, with **`S`** $=\sigma$, **`J`** $= j$, and **`P`** $= 1,\ldots,\lceil \#\mathcal{S} / \left(2.5\times 10^5\right)\rceil$. We then generated a corresponding set of Paramotopy input files, and determined the subset of bistable points in each sample file via the workflow in Fig. A.

These bistable subsets are indicated thus in the corresponding posreal counts files, which can be found in **Dataset 4**x**.2**, for $x = \mathbf{H}, \ldots, \mathbf{U}$, as

`sigma[S]_log_link[J]_part[P]_posreal_run0`

with the same values of `S`, `J`, and `P` as given above. These posreal counts files were then aggregated over $\texttt{P} = 1, \ldots, \lceil \#\mathcal{S} / (2.5 \times 10^5) \rceil$ (cf. footnote 14), generating the aggregate posreal counts file (**Dataset 4**x**.2**, for $x = \mathbf{H}, \ldots, \mathbf{U}$)

`sigma[S]_log_link[J]_posreal_run0`

The bistable points in each of these aggregate posreal counts files were then extracted with `count_bistable.py` (cf. footnote 15), to generate the bistable points file (**Dataset 4**x**.2**, for $x = \mathbf{H}, \ldots, \mathbf{U}$)

`sigma[S]_log_link[J]_multistable`

which was then concatenated with (1) the bistable points file with which the initial connectivity graph was constructed (**Dataset 4**x**.2**, for $x = \mathbf{H}, \ldots, \mathbf{U}$; see §5, *Building the initial connectivity graphs*),

`sigma[S]_combined_vegas_N1_log_multistable`

and (2) the bistable points files obtained from previous iterations of the refinement (**Dataset 4**x**.2**, for $x = \mathbf{H}, \ldots, \mathbf{U}$),

`sigma[S]_log_link[K]_multistable`

with $\texttt{K} = 0, \ldots, j-1$, to generate the aggregate bistable points file (**Dataset 4**x**.2**, for $x = \mathbf{H}, \ldots, \mathbf{U}$)

`sigma[S]_combined_log_link[J]_multistable`

This file was then passed into `runLogConnectivity.m` to complete the j-th iteration of the refinement, the output of which was a new MAT file summarizing the refined connectivity graph (**Dataset 6**),

`sigma[S]_data/sigma[S]_log_link[J]_connect.mat`

Parsing these MAT files with `reportConnectivityData.m`, we found that two iterations of refinement were sufficient to generate a single-component graph for each value of σ (Table C), thus bolstering our hypothesis that the bistable region is indeed connected for all σ.

6 Locating blinking points within the connectivity graph

Here, we describe the analyses that were undertaken to identify blinking parameter points, certify their associated proper solutions, and locate them within the connectivity graphs described in the previous section.

First, we implemented a Python script, `find_blinking_points.py`, which identifies the subset of bistable points at each value of σ that become monostable at some larger value of σ, then identifies the component to which each such point belongs in the corresponding initial connectivity graph. More specifically, the script reads, for each value of σ in Eq. 15 greater than 2.5, the aggregate bistable points file (cf. footnote 15) (**Dataset 4**x**.2**, for $x = \mathbf{K}, \ldots, \mathbf{U}$)

`sigma[S]_N1_log_multistable`

while, for $\sigma = 1.5, 2.0, 2.5$, the script reads the aggregate bistable points file[16] (**Dataset 4**x**.2**, for $x = \mathbf{H}, \mathbf{I}, \mathbf{J}$)

`sigma[S]_N1_log_4M_multistable`

which only contains the subset of bistable points among the 4×10^6 ILR-sampled parameter points from $\mathcal{H}$ at which a solution set was obtained from every value of σ in Eq. 15. The script then identifies, for each $\sigma = a$, every bistable point that does not feature among the bistable points at some $\sigma = b > a$, where a and b are among the values in Eq. 15. Upon loading the MAT files containing the initial connectivity graphs for $\sigma \geq a$ (see §5, *Building the initial connectivity graphs*), the script then identifies those subsets of the identified blinking points satisfying the following properties (see the Results for details):

- Falls within a non-largest component in the initial connectivity graph for $\sigma = a$ (Table 2, "# BPSC").
- Does not fall within the largest component in any of the initial connectivity graphs for $\sigma > a$ (Table 2, "# BPNL").
- Is monostable at $\sigma = 500$ (Table 2, "# BPAM").

Second, we sought to verify that blinking is a genuine property of these parameter points, by certifying each of their proper solutions with alphaCertified. We implemented a Python script, `find_blinking_solutions.py`, which runs through the blinking points identified with `find_blinking_points.py` and extracts the proper solution sets associated with each blinking

[16] This aggregate bistable points file was generated by running `count_bistable.py` (cf. footnote 15) on the aggregate posreal counts file

`sigma[S]_N1_log_4M_posreal_total`

which was generated by concatenating the posreal counts files

`sigma[S]_N1_log_part[P]_posreal_run[R]`

with `S` $= \sigma$, over `P` $= 1, \ldots, 4$ and `R` $= 0, \ldots, 3$.

point, for each value of σ listed in Eq. 11. We then performed the same procedure for certification as described above (§3), and were able to certify every proper solution associated with each blinking point at each value of σ. All composite output files generated from this procedure are given in **Dataset 5Y**.

7 Computing the K-fold visibility ratio

Here, we describe our implementation of the K-fold visibility ratio computation. As described in the Results, we estimated the 10-fold visibility ratio, $\text{vis}(\mathcal{M}_\sigma, K=10)$, of the bistable region at a given value of σ by randomly choosing, without replacement, $M = 20000$ pairs of parameter points,

$$\left(\theta^{(1)}, \mu^{(1)}\right), \dots, \left(\theta^{(M)}, \mu^{(M)}\right) \in \widehat{\mathcal{M}}_\sigma \times \widehat{\mathcal{M}}_\sigma,$$

where $\widehat{\mathcal{M}}_\sigma$ is a bistable set gathered through ILR sampling from $\mathcal{H}$, then computing the estimator,

$$\widehat{\text{vis}}\left(\mathcal{M}_\sigma, K\right) = \frac{1}{M} \sum_{j=1}^{M} \nu_{10}\left(\theta^{(j)}, \mu^{(j)}\right). \tag{A}$$

To perform this estimation, we implemented a MATLAB script, `getConvexitySamples.m`, that randomly chooses 20000 pairs of points from a bistable set for a given value of σ, and generates $MK = 2 \times 10^5$ parameter points, as described in the Results. Each bistable set was read from the bistable points file (cf. footnote 15) (**Dataset 4x.2**, for $x = \mathbf{I}, \dots, \mathbf{U}$),

`sigma[S]_N1_log_multistable`

with `S` $= \sigma$. Each resulting sample of 2×10^5 parameter points was then written to a sample file, `sample_convexity_sigma[S]`, again with `S` $= \sigma$ (**Dataset 2Q**). The points in each sample file were ordered such that the 10 points sampled between each pair were written consecutively, so that $\nu_{10}\left(\theta^{(j)}, \mu^{(j)}\right)$ could be determined by simply looking up the solutions for points $10\left(j-1\right), \dots, 10j-1$ in the sample. We then applied the workflow in Fig. A to obtain the bistable subset in each sample at the corresponding value of σ. The resulting proper solutions file for $\sigma = 2.0$ can be found in **Dataset 4I.10**, at the path

`sigma2_convexity_solutions_run0`

and the resulting proper solutions file for $\sigma > 2.0$ can be found in **Dataset 4x.9**, for $x = \mathbf{J}, \dots, \mathbf{U}$, at the path

`sigma[S]_convexity_solutions_run0`

with `S` $= \sigma$. Moreover, the corresponding final posreal counts file can be found in **Dataset 4x.2**, for $x = \mathbf{I}, \dots, \mathbf{U}$, at the path

`sigma[S]_convexity_posreal_run0`

These final posreal counts files were parsed to compute the estimator in Eq. A for each value of σ. This was implemented in a Python script, `plot_fig4A_visratios.py`, which also plots the estimates to produce Fig. 4A.

8 Plotting the figures in the main text

Finally, we also provide the scripts with which we generated Figs. 3 through 9.

- **Fig. 3 (`plot_fig3_volumes.py`).** For each value of σ in Eq. 11, the bistable volume was estimated by counting the proportion of parameter points in the aggregate posreal counts file (**Dataset 4**x**.2**, for $x = \mathbf{A}, \mathbf{H}, \ldots, \mathbf{U}$)

  `sigma[S]_N1_log_posreal_total`

  with `S` $= \sigma$, having more than one positive real proper solution. Confidence intervals were estimated as described in the Materials and Methods.

- **Fig. 4A (`plot_fig4A_visratios.py`).** For each value of σ in Eq. 11 except for $\sigma = 1.0$ and $\sigma = 1.5$, the 10-fold visibility ratio was estimated by parsing the posreal counts file (**Dataset 4**x**.2**, for $x = \mathbf{I}, \ldots, \mathbf{U}$)

  `sigma[S]_convexity_posreal_run0`

  with `S` $= \sigma$, and computing the estimator in Eq. A, as described above. Confidence intervals were estimated as described in the Materials and Methods.

- **Fig. 4B (`plot_fig4B_schematic.py`).** The 10-fold visibility ratios of the three regions were estimated in the same way as with the bistable region. For each value of a/R (between 0 and 0.5 for the ribbon and star, between 0 and 1 for the annulus), we randomly generated 20000 points within each region with a Monte Carlo procedure, randomly chose 20000 pairs of the points in each region, and determined each pair to be mutually visible if 10 evenly spaced points along the straight line between them lay within the region. The proportion of mutually visible pairs—the estimated visibility ratio—was then plotted over a mesh of values for a/R.

- **Fig. 5 (`plot_fig5_curves.py`).** We generated each plot of the two curves, for $\Phi_1(u, v) = 0$ and $\Phi_2(u, v) = 0$, with the `plot_implicit()` and `pyplot.contour()` methods in the Python packages SymPy (version 1.1.1) and Matplotlib (version 2.1.0) [9, 10]; the point(s) of intersection were determined by parsing the relevant proper solutions file (see below). To find the four parameter points whose corresponding curves are plotted, we parsed the aggregate posreal counts files (**Dataset 4**x**.2**, for $x = \mathbf{K}, \mathbf{L}, \mathbf{M}, \mathbf{N}, \mathbf{O}$)

  `sigma[S]_N1_log_posreal_total`

  and bistable points files (**Dataset 4**x**.2**, for $x = \mathbf{K}, \mathbf{L}, \mathbf{M}, \mathbf{N}, \mathbf{O}$; cf. footnote 15)

  `sigma[S]_N1_log_multistable`

  for `S` $= \sigma = 3.0, 4.0, 5.0, 7.0, 10$, from which we identified four non-intersecting subsets of parameter points in $\mathcal{H}$:

– Points that exhibit monostability at all five values of σ;

– Points monostable at $\sigma = 3.0$ that become bistable at $\sigma = a > 3.0$ and remains so for $a \leq \sigma \leq 10$;

– Points monostable at $\sigma = 3.0$ that become bistable at $\sigma = a > 3.0$, revert to monostable at $\sigma = b > a$, and remains so for $b \leq \sigma \leq 10$; and

– Points monostable at $\sigma = 3.0$ that become bistable $\sigma = a > 3.0$, reverts to monostable at $\sigma = b > a$, reverts to bistable at $\sigma = c > b$, and remains so for $c \leq \sigma \leq 10$.

We then randomly chose 200 combinations of four parameter points, one from each category, and plotted the corresponding curves and their intersection points at each of the five values of σ, as indicated above. The intersection points were obtained by parsing the corresponding 16 proper solutions files (**Dataset 4**x**.4**, for $x =$ **K**, **L**, **M**, **N**, **O**)

`sigma[S]_N1_log_part[P]_solutions_run[R]`

with `P` running over $1, \ldots, 4$ and `R` over $0, \ldots, 3$. The particular choice of the four parameter points given in Fig. 5 was made for cosmetic reasons; subsequently, an additional option for generating the plots corresponding to these points (and thus replicate Fig. 5) was added to `plot_fig5_curves.py`.

- **Fig. 6 (`plot_fig6_strong_irrev_volumes.py`).** For each value of σ in Eq. 11, the bistable volume was estimated by counting the proportion of parameter points in the aggregate posreal counts file (**Dataset 4**x**.2**, for $x =$ **A**, **H**, $\ldots$, **X**)

  `sigma[S]_michaelis-menten_N1_log_posreal_total`

  with `S` $= \sigma$, having more than one positive real proper solution. Confidence intervals were estimated as described in the Materials and Methods.

- **Fig. 7A (`plot_fig7A_projections.py`).** For each indicated value of σ (top right in each plot), the projection were generated by parsing the bistable points file (**Dataset 4**x**.2**, for $x =$ **J**, $\ldots$, **U**; cf. footnote 15)

  `sigma[S]_N1_log_multistable`

  with `S` $= \sigma$, and picking out the fifth and sixth coordinates—corresponding to ε_2 and φ_0—in each point. The estimated multiplicative bound (bottom right in each plot) was computed as the maximum value of $\varepsilon_2\varphi_0$ over all points in each file.

- **Fig. 7B (`plot_fig7B_schematic.nb`).** This plot was generated with Mathematica (version 10.2.0.0), via the command

```
RegionPlot3D[Log[10, x] + Log[10, y] < Log[10, 7] && Log[10, z] > 1 - 0.2 * 2, {x, 0.1, 10},
↪  {y, 0.1, 1}, {z, 0.1, 1}, ...]
```

so as to illustrate the disparity in normalized volume between a one-dimensional region occupying 20% of the (logarithmic) interval $[-1, 1]$ and a three-dimensional region exhibiting a parametric tradeoff, $\varepsilon_2\varphi_0 < K = 7.0$.

- **Fig. 8** (`plot_fig8_outer_volumes.py`)**.** For each value of σ in Eqs. 11 and 20 and $p = 2, 3, 4, 5$, the bistable volume, $\widehat{V}_{\sigma,p}$, was estimated by counting the proportion of parameter points in the aggregate posreal counts file (**Dataset 4**x**.2**, for $x = \mathbf{A}, \ldots, \mathbf{U}$)

  `sigma[S]_N[P]_log_posreal_total`

  with `S` $= \sigma$ and `P` $= p$, having more than one positive real proper solution. Confidence intervals were estimated as described in the Materials and Methods.

- **Fig. 9** (`plot_fig9_strong_irrev_outer_volumes.py`)**.** For each value of σ in Eqs. 11 and 20 and $p = 2, 3, 4, 5$, the bistable volume, $\widehat{V}^*_{\sigma,p}$, was estimated by counting the proportion of parameter points in the aggregate posreal counts file (**Dataset 4**x**.2**, for $x = \mathbf{A}, \ldots, \mathbf{U}$)

  `sigma[S]_michaelis-menten_N[P]_log_posreal_total`

  with `S` $= \sigma$ and `P` $= p$, having more than one positive real proper solution. Confidence intervals were estimated as described in the Materials and Methods.

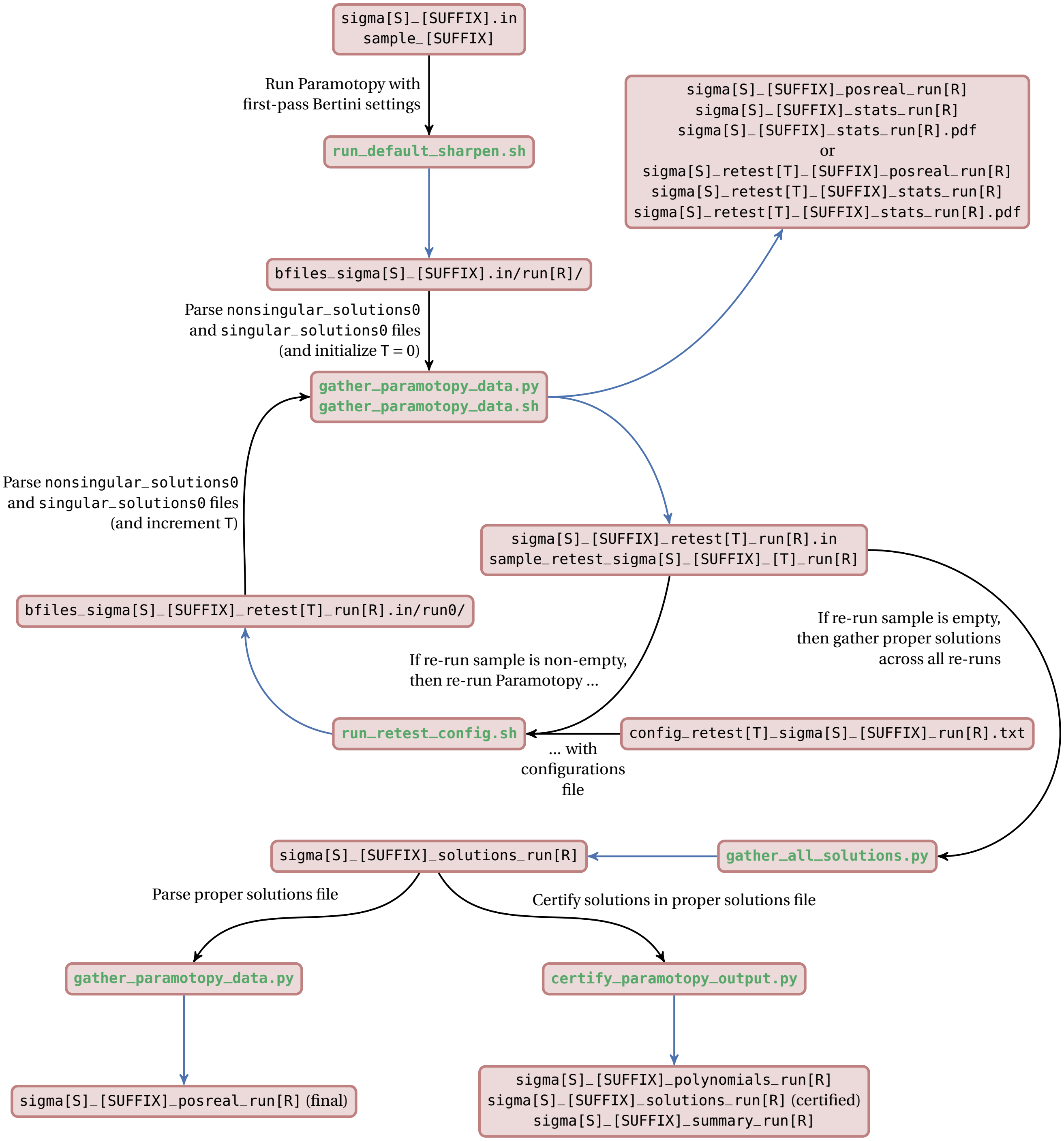

Figure A: **Workflow for computing and parsing solutions with Paramotopy.** Each black arrow connects a collection of input files or directories to the script to which they are passed; each blue arrow connects a script to its collection of output files or directories. Certain aspects of the workflow (e.g., path failure resolution via the Paramotopy interface) are omitted for clarity.

Sample	Seed	Dataset	Paths to sample files
10^7 in $\mathcal{H}$ (ILR)	4236	**2A**	sample_N1_[J]_log, $J = 1,\ldots,40$
10^6 in $\mathcal{H}_2$ (ILR)	8990	**2A**	sample_N2_[J]_log, $J = 1,2,3,4$
10^6 in $\mathcal{H}_3$ (ILR)	8527	**2A**	sample_N3_[J]_log, $J = 1,2,3,4$
10^6 in $\mathcal{H}_4$ (ILR)	5700	**2A**	sample_N4_[J]_log, $J = 1,2,3,4$
10^6 in $\mathcal{H}_5$ (ILR)	2289	**2A**	sample_N5_[J]_log, $J = 1,2,3,4$
6×10^6 in $\mathcal{H}$ (VEGAS, $\sigma \leq 2.0$, $t = 1$)	6754	**2O**	sample_vegas_N1_log_lowsigma_[J], $J = 1,\ldots,24$
4603 in $\mathcal{H}$ (linking, $\sigma = 1.5$, $j = 0$)	2725	**2P**	sample_log_link_sigma1.5_0_1
32012 in $\mathcal{H}$ (linking, $\sigma = 2.0$, $j = 0$)	4030	**2P**	sample_log_link_sigma2_0_1
86996 in $\mathcal{H}$ (linking, $\sigma = 2.5$, $j = 0$)	4030	**2P**	sample_log_link_sigma2.5_0_1
143550 in $\mathcal{H}$ (linking, $\sigma = 3.0$, $j = 0$)	3420	**2P**	sample_log_link_sigma3_0_1
252719 in $\mathcal{H}$ (linking, $\sigma = 4.0$, $j = 0$)	8646	**2P**	sample_log_link_sigma4_0_[K], $K = 1,2$
345683 in $\mathcal{H}$ (linking, $\sigma = 5.0$, $j = 0$)	6972	**2P**	sample_log_link_sigma5_0_[K], $K = 1,2$
476828 in $\mathcal{H}$ (linking, $\sigma = 7.0$, $j = 0$)	3071	**2P**	sample_log_link_sigma7_0_[K], $K = 1,2$
592409 in $\mathcal{H}$ (linking, $\sigma = 10$, $j = 0$)	529	**2P**	sample_log_link_sigma10_0_[K], $K = 1,2,3$
704916 in $\mathcal{H}$ (linking, $\sigma = 15$, $j = 0$)	6853	**2P**	sample_log_link_sigma15_0_[K], $K = 1,2,3$
760821 in $\mathcal{H}$ (linking, $\sigma = 20$, $j = 0$)	5326	**2P**	sample_log_link_sigma20_0_[K], $K = 1,2,3,4$
872493 in $\mathcal{H}$ (linking, $\sigma = 50$, $j = 0$)	351	**2P**	sample_log_link_sigma50_0_[K], $K = 1,2,3,4$
911144 in $\mathcal{H}$ (linking, $\sigma = 100$, $j = 0$)	7670	**2P**	sample_log_link_sigma100_0_[K], $K = 1,2,3,4$
936037 in $\mathcal{H}$ (linking, $\sigma = 200$, $j = 0$)	7670	**2P**	sample_log_link_sigma200_0_[K], $K = 1,2,3,4$
947351 in $\mathcal{H}$ (linking, $\sigma = 500$, $j = 0$)	9910	**2P**	sample_log_link_sigma500_0_[K], $K = 1,2,3,4$
450 in $\mathcal{H}$ (linking, $\sigma = 1.5$, $j = 1$)	248	**2P**	sample_log_link_sigma1.5_1_1
1300 in $\mathcal{H}$ (linking, $\sigma = 2.0$, $j = 1$)	2089	**2P**	sample_log_link_sigma2_1_1
2279 in $\mathcal{H}$ (linking, $\sigma = 2.5$, $j = 1$)	7428	**2P**	sample_log_link_sigma2.5_1_1
3230 in $\mathcal{H}$ (linking, $\sigma = 3.0$, $j = 1$)	6121	**2P**	sample_log_link_sigma3_1_1
5730 in $\mathcal{H}$ (linking, $\sigma = 4.0$, $j = 1$)	2263	**2P**	sample_log_link_sigma4_1_1
6237 in $\mathcal{H}$ (linking, $\sigma = 5.0$, $j = 1$)	9847	**2P**	sample_log_link_sigma5_1_1
6757 in $\mathcal{H}$ (linking, $\sigma = 7.0$, $j = 1$)	4830	**2P**	sample_log_link_sigma7_1_1
2267 in $\mathcal{H}$ (linking, $\sigma = 10$, $j = 1$)	9352	**2P**	sample_log_link_sigma10_1_1
12156 in $\mathcal{H}$ (linking, $\sigma = 15$, $j = 1$)	1405	**2P**	sample_log_link_sigma15_1_1
13498 in $\mathcal{H}$ (linking, $\sigma = 20$, $j = 1$)	3210	**2P**	sample_log_link_sigma20_1_1
4123 in $\mathcal{H}$ (linking, $\sigma = 50$, $j = 1$)	4196	**2P**	sample_log_link_sigma50_1_1
15436 in $\mathcal{H}$ (linking, $\sigma = 100$, $j = 1$)	3537	**2P**	sample_log_link_sigma100_1_1
16644 in $\mathcal{H}$ (linking, $\sigma = 200$, $j = 1$)	5146	**2P**	sample_log_link_sigma200_1_1
4579 in $\mathcal{H}$ (linking, $\sigma = 500$, $j = 1$)	3211	**2P**	sample_log_link_sigma500_1_1
2×10^5 in $\mathcal{H}$ (convexity, $\sigma = 2.0$)	8148	**2Q**	sample_convexity_sigma2
2×10^5 in $\mathcal{H}$ (convexity, $\sigma = 2.5$)	2142	**2Q**	sample_convexity_sigma2.5
2×10^5 in $\mathcal{H}$ (convexity, $\sigma = 3.0$)	9750	**2Q**	sample_convexity_sigma3
2×10^5 in $\mathcal{H}$ (convexity, $\sigma = 4.0$)	6971	**2Q**	sample_convexity_sigma4
2×10^5 in $\mathcal{H}$ (convexity, $\sigma = 5.0$)	4058	**2Q**	sample_convexity_sigma5
2×10^5 in $\mathcal{H}$ (convexity, $\sigma = 7.0$)	2277	**2Q**	sample_convexity_sigma7
2×10^5 in $\mathcal{H}$ (convexity, $\sigma = 10$)	8813	**2Q**	sample_convexity_sigma10
2×10^5 in $\mathcal{H}$ (convexity, $\sigma = 15$)	3367	**2Q**	sample_convexity_sigma15
2×10^5 in $\mathcal{H}$ (convexity, $\sigma = 20$)	8014	**2Q**	sample_convexity_sigma20
2×10^5 in $\mathcal{H}$ (convexity, $\sigma = 50$)	3329	**2Q**	sample_convexity_sigma50
2×10^5 in $\mathcal{H}$ (convexity, $\sigma = 100$)	9532	**2Q**	sample_convexity_sigma100
2×10^5 in $\mathcal{H}$ (convexity, $\sigma = 200$)	1031	**2Q**	sample_convexity_sigma200
2×10^5 in $\mathcal{H}$ (convexity, $\sigma = 500$)	624	**2Q**	sample_convexity_sigma500

Table A: **Seeds used to initialize the MATLAB pseudo-random number generator for sampling.** The first column specifies how the sample was generated (number of points, domain, and method), the second specifies the seed, and the third and fourth specify the dataset and path at which the sample is located.

σ \ t	Dataset	1	2	3	4	5	6
2.5	**2C**	3939	8190	6690	8744	5326	3380
3.0	**2D**	7919	9149	4185	5719	5817	4819
4.0	**2E**	5045	9475	8972	9675	8673	9801
5.0	**2F**	2225	5625	4097	5218	5716	4765
7.0	**2G**	4598	6587	2486	3771	4307	9879
10	**2H**	4080	9576	6288	2352	5579	5448
15	**2I**	7161	4770	2976	5740	3400	385
20	**2J**	6692	7586	719	5668	1833	248
50	**2K**	3514	5182	8587	3616	7722	272
100	**2L**	5190	3359	4708	3522	3935	8712
200	**2M**	4841	9782	4802	9344	8406	8393
500	**2N**	9078	797	6687	2869	7261	7730

Table B: **Seeds used to initialize the MATLAB pseudo-random number generator for VEGAS sampling.** The first column specifies the value of σ for each row; the second column specifies the dataset in which all corresponding samples are located; and each subsequent column corresponds to an iteration number, $t = 1, \ldots, 6$. As described in §4 (see also the Results), each sample consists of $N = 10^6$ points, and was written to four sample files, `sample_vegas_N1_log_sigma[S]_[T]_[P]`, with `S` $= \sigma$, `T` $= t - 1$, and `P` $= 1, \ldots, 4$. The seed used to generate the additional single-iteration VEGAS sample for $\sigma < 2.5$ is given in Table A.

	σ	$\#\widehat{\mathcal{M}}_\sigma$	# components	$\#C_1$	$\#C_2$	# singletons	$\#C_1/\#\widehat{\mathcal{M}}_\sigma$
Iteration 1	1.5	183713	20	183694	1	19	> 99.9%
	2.0	1513383	42	1513340	2	39	> 99.9%
	2.5	2543059	70	2542988	2	67	> 99.9%
	3.0	2849291	91	2849197	2	86	> 99.9%
	4.0	3184488	155	3184323	3	145	> 99.9%
	5.0	3396191	160	3396025	2	152	> 99.9%
	7.0	3623448	184	3623252	3	172	> 99.9%
	10	3828193	237	3827937	4	218	> 99.9%
	15	3975467	327	3975110	3	297	> 99.9%
	20	4063798	348	4063423	2	319	> 99.9%
	50	4237924	424	4237450	4	382	> 99.9%
	100	4292358	401	4291920	3	364	> 99.9%
	200	4336290	428	4335835	3	400	> 99.9%
	500	4343449	465	4342941	4	429	> 99.9%
	σ	$\#\widehat{\mathcal{M}}_\sigma$	# components	$\#C_1$	$\#C_2$	# singletons	$\#C_1/\#\widehat{\mathcal{M}}_\sigma$
Iteration 2	1.5	184109	1	184109	n/a	0	100%
	2.0	1514549	1	1514549	n/a	0	100%
	2.5	2545157	1	2545157	n/a	0	100%
	3.0	2852239	1	2852239	n/a	0	100%
	4.0	3189724	1	3189724	n/a	0	100%
	5.0	3401875	1	3401875	n/a	0	100%
	7.0	3629666	1	3629666	n/a	0	100%
	10	3830243	1	3830243	n/a	0	100%
	15	3986582	1	3986582	n/a	0	100%
	20	4076178	1	4076178	n/a	0	100%
	50	4241686	1	4241686	n/a	0	100%
	100	4306436	1	4306436	n/a	0	100%
	200	4351496	1	4351496	n/a	0	100%
	500	4347535	1	4347535	n/a	0	100%

Table C: **Details of the refined connectivity graphs.** This table follows the same format as Table 1.

References.

[1] Daniel J. Bates, Jonathan D. Hauenstein, Andrew J. Sommese, and Charles W. Wampler. *Numerically solving polynomial systems with Bertini*. Vol. 25. SIAM, 2013.

[2] Daniel J. Bates, Silviana V. Amethyst, and Matt Niemerg. "`Paramotopy`: parameter homotopies in parallel". In: *Mathematical Software – ICMS 2018*. Vol. 10931. 2018, pp. 28–35.

[3] Silviana V. Amethyst, Matthew E. Niemerg, and Daniel J. Bates. *Paramotopy: parallel parameter homotopy via Bertini*. English. Version 1.0.3.5. Manual. Aug. 2015. URL: `http://www.paramotopy.com/resources/infographics/general/paramotopy_manual.pdf`.

[4] Jonathan D. Hauenstein and Frank Sottile. "Algorithm 921: alphaCertified: certifying solutions to polynomial systems". In: *ACM Trans Math Softw* 38.4 (2012), p. 28.

[5] Jonathan D. Hauenstein. *alphaCertified*. English. Version 1.3. Manual. Sept. 2011. URL: `http://www.math.tamu.edu/~sottile/research/stories/alphaCertified/Download/V13/alphaCertified.pdf`.

[6] Shai Bagon. *MATLAB class for computing approximate nearest neighbors*. Mar. 2012. URL: `http://www.wisdom.weizmann.ac.il/~bagon/matlab.html`.

[7] Sunil Arya, David M. Mount, Nathan S. Netanyahu, Ruth Silverman, and Angela Y. Wu. "An optimal algorithm for approximate nearest neighbor searching in fixed dimensions". In: *J ACM* 45.6 (1998), pp. 891–923.

[8] David M. Mount and Sunil Arya. *ANN: A library for approximate nearest neighbor searching*. Version 1.1.1. Aug. 2006. URL: `http://www.cs.umd.edu/~mount/ANN/`.

[9] John D. Hunter. "Matplotlib: A 2D graphics environment". In: *Comput Sci Eng* 9.3 (2007), pp. 90–95.

[10] Aaron Meurer et al. "SymPy: symbolic computing in Python". In: *PeerJ Comput Sci* 3 (2017), e103.
