# Supplementary material for: Robustness and parameter geography in post-translational modification systems
Source: PLoS Comput Biol. 2020 May 4;16(5):e1007573. doi: 10.1371/journal.pcbi.1007573 (PMC7224580; doi:10.1371/journal.pcbi.1007573)
Supplement: Attachment — Submitted filename: response-to-reviewers.pdf [file pcbi.1007573.s005.pdf]

PCOMPBIOL-D-19-02084
"*Robustness and parameter geography in post-translational modification systems*"
by Nam, Gyori, Amethyst, Bates, Gunawardena

General remarks.

We are grateful to the three reviewers for their efforts on our behalf and their questions and suggestions and especially for pointing out further directions to explore. We hope to have addressed the comments as carefully as we can, as detailed below. Unless otherwise stated, line numbers refer to the revised and marked-up version of the paper.

Detailed responses.

Reviewer #1.

We are pleased that the reviewer found that "*the methods and analyses seem sound*" and that these contributions were "*valued*".

1. **"I still find it very difficult to really understand the implications. What are really the biologically significant findings? ... I think the authors need to work on interpreting and also presenting their work such that it is more clear what the results mean in terms of signaling biology."**

- We believe the reviewer has already pointed out one of our significant findings: the importance of assuming realistic enzyme mechanisms for drawing conclusions about biological robustness. As we noted in the paper, there is widespread dependence in the literature on the Michaelis-Menten enzyme mechanism. The fact that this is unrealistic because of product rebinding has been known for years, with apparently no effect. Here, we show in a compelling way why it can be so misleading. To say, as the reviewer does, that "*bistability is less likely to occur*", does not draw out the biological implications. Our results show that the probability of bistability reduces from 20% under the strongly irreversible Michaelis-Menten mechanism to only 1.1% for any realistic mechanism. The former figure suggests that bistability may be quite robust to change in parameter values; the latter figure does not support this conclusion at all. We believe this is a significant contribution to understanding the robustness of PTM systems. To address the reviewer's concern, we have added a paragraph to the Discussion (page 22, lines 913-917) which explains more clearly the significance of our results for biological robustness.

  The reviewer described the finding above as the "*major contribution*" of the paper, after its "*algorithmic approach*". We would also draw attention to the two mathematical conjectures and question which we state in the Discussion. We believe these are also important contributions which suggest new directions of research into biochemical networks.

2. **"Can we understand it mechanistically, why bistability requires that substrate and enzyme concentrations are like described in the paper?"**

- We were uncertain as to what precisely the reviewer is referring to here but the question seems to concern the existence of a threshold which must be exceeded for bistability to occur, as described in our first Conjecture in the Discussion section. We agree that it is interesting to ask what underlies this threshold "*mechanistically*" but this seems beyond the scope of the present

paper. The contribution of the paper has been to reveal the existence of the threshold and to conjecture the form it should take for more general systems. We feel that we must leave it to subsequent work to explain the threshold in more detail.

Reviewer #2.

We are pleased that the reviewer found that "*overall, the paper is well written*", "*overall this is a good balance between application, theory and computation*" and recommends "*it to be published*".

3. **"The visibility ratio sounds like it is approximating geodesics. Can numerical algebraic geometry approximate this variety in parameter space, and provide some indication of the degree of this "visibility" curve?"**

- We are not sure what the reviewer has in mind here. The visibility ratio is a number which measures how close the region is to being convex. There is a concept of "geodesic convexity", which could be considered for the surface of the region, with the induced Riemannian metric coming from the standard metric on the ambient parameter space. However, this is a different idea to the normal convexity of the region, for which the visibility ratio was calculated. As for the second question, we are using numerical algebraic geometry to identify points in the region and to estimate the visibility ratio. We are not sure what "visibility curve" the reviewer has in mind here, whose degree is being sought, as we have not seen this concept in the literature.

4. **"The ``grammar" sounds similar to studying the graph rather than the resulting equations, which is a feature of chemical reaction network theory. It would be useful to explain the connection when presenting chemical Eqs (1). For example, this grammar sounds similar to generalized mass action kinetics."**

- Indeed, the grammar is formulated in the linear framework, which, as we stated (previous line 122), is "graph-based". The linear framework is related to chemical reaction network theory, as noted in the original publication (citation 69), but the corresponding graphs have different interpretations. We feel it would be too much of a digression to explain this in the text but <u>we have reiterated the graph aspects in the relevant paragraph (lines 134-135)</u>. There is no connection to generalised mass-action kinetics and ordinary mass action is used throughout.

5. **"The authors should discuss how their parameter geography compares to the work below, and whether the methods described below could provide insight to prove their conjectures:"**

- Thank you! We cited Conradi, Feliu et al previously (citation 58, now citation 56) but we had not taken note of papers by Joshi & Shiu and Harrington, Mehta et al. We hope to have identified these correctly from what the reviewer writes as new citations 60 and 67, respectively. We greatly appreciate these references. <u>We have mentioned these works in the Introduction (lines 75-78) and added a paragraph to the Discussion (lines 961-966)</u>, in which we review how these and other approaches may provide insight into our conjectures.

6. **"For the parameter values that could not be certified, are these near the discriminant locus?"**

- There are different types of "discriminant locus" depending on what kind of non-generic behaviour is being discriminated. Harrington, Mehta et al discuss some of the choices (citation

67, suggested by the reviewer) . If “discriminant locus” means those parameter values for which some steady state has a singular Jacobian, then the answer is yes. We pointed out in the Methods that “*Manual inspection of the alphaCertified output suggests that the uncertifiable solutions are in fact close to singular*” (original lines 1172-1173, new lines 1187-1188).

7. **“It would be helpful to have more details in table captions.”**

- Thank you. We have included more details in the table captions (page 43).

Reviewer #3.

We are pleased that the reviewer found that “*the setup for the computational experiments described in the paper appear to be reasonable while the scale of these computations is quite remarkable*”.

8. **“My only comment on the paper is in regards to decomposing the parameter space: Was there an attempt to symbolically compute the discriminant polynomial of (10)? Or compute it using numerical algebraic geometry? What is its degree?”**

- The short answers to the first two questions are no, we did not. The longer answers relate to the point made in response 6 above, about the nature of the “discriminant polynomial”. For discriminating singular solutions, the corresponding “locus” in parameter space is an algebraic variety for which a degree may be calculated. However, for the present paper, we are interested in the number of positive, real roots and it is not clear to us whether the corresponding “discriminant locus” would even be an algebraic variety. If it is not, then we cannot compute it symbolically or determine its degree. It may be possible to approximate it numerically, as the reviewer suggests. Something like this was done by Harrington, Mehta et al in the citation (67) suggested by Reviewer #2, but their methods appear limited to parameter spaces of low dimension (Figure 3 of citation 67 is for a parameter space of dimension 2). Accordingly, we feel that exploring such an alternative approach must be left to subsequent studies. We thank the reviewer for suggesting this direction.
